# Using Functional Data Analysis Models to Estimate Future Time Trends in Age-Specific Breast Cancer Mortality for the United States and England–Wales

**DOI:** 10.2188/jea.JE20090072

**Published:** 2010-03-05

**Authors:** Bircan Erbas, Muhammed Akram, Dorota M Gertig, Dallas English, John L. Hopper, Anne M Kavanagh, Rob Hyndman

**Affiliations:** 1 School of Public Health, La Trobe University, Bundoora, 3086 Australia; 2 Business & Economic Forecasting Unit, Monash University, Clayton, 3800, Australia; 3 Victoria Cytology Service Inc, Carlton, 3053 Australia; 4 Cancer Epidemiology Centre, The Cancer Council Victoria, Carlton 3053 Australia; 5 Centre for MEGA Epidemiology, The University of Melbourne, Parkville 3053 Australia; 6 Key Centre for Women’s Health in Society, School of Population Health, The University of Melbourne, Parkville, 3053 Australia

**Keywords:** breast cancer, forecasting, functional-data-analysis models, mortality trends

## Abstract

**Background:**

Mortality/incidence predictions are used for allocating public health resources and should accurately reflect age-related changes through time. We present a new forecasting model for estimating future trends in age-related breast cancer mortality for the United States and England–Wales.

**Methods:**

We used functional data analysis techniques both to model breast cancer mortality-age relationships in the United States from 1950 through 2001 and England–Wales from 1950 through 2003 and to estimate 20-year predictions using a new forecasting method.

**Results:**

In the United States, trends for women aged 45 to 54 years have continued to decline since 1980. In contrast, trends in women aged 60 to 84 years increased in the 1980s and declined in the 1990s. For England–Wales, trends for women aged 45 to 74 years slightly increased before 1980, but declined thereafter. The greatest age-related changes for both regions were during the 1990s. For both the United States and England–Wales, trends are expected to decline and then stabilize, with the greatest decline in women aged 60 to 70 years. Forecasts suggest relatively stable trends for women older than 75 years.

**Conclusions:**

Prediction of age-related changes in mortality/incidence can be used for planning and targeting programs for specific age groups. Currently, these models are being extended to incorporate other variables that may influence age-related changes in mortality/incidence trends. In their current form, these models will be most useful for modeling and projecting future trends of diseases for which there has been very little advancement in treatment and minimal cohort effects (eg. lethal cancers).

## INTRODUCTION

Breast cancer is the most commonly diagnosed cancer in women worldwide.^1^ In the United States and United Kingdom, breast cancer is the second highest cause of cancer death in women.^2^^,^^3^ Therefore, accurate projections of mortality/incidence from breast cancer are important for planning future public health policy and resource allocation. In particular, accurate age-specific projections of mortality from breast cancer are essential for assessing cancer control programs such as mammographic screening. Screening, combined with improvements in treatment options, influences the mortality and incidence patterns for women of different ages.^4^^,^^5^ Because trends in breast cancer mortality and incidence vary substantially with age, it is important that predictions accurately consider and reflect these variations.

There are noticeable differences in breast cancer mortality patterns over time by age across different countries. In the United Kingdom, breast cancer mortality has decreased substantially for women between the ages of 55 and 69 years, as compared with those aged 50 to 54 years.^6^ Conversely, in the United States, breast cancer mortality for white women has declined more rapidly for those younger than 50 years than for those who are older.^5^ A decrease in mortality for women aged 30 to 49 years has been observed in a number of European countries that lack an organized nationwide screening program.^4^ Mortality among women older than 65 years has continued to increase in many countries, regardless of screening or advances in treatment.^7^^,^^8^

Differences in age-related mortality trends between countries may reflect differences in mammographic screening policies and age-related differences in the uptake of treatment options (such as tamoxifen). To take one example, there is triennial screening of 50- to 70-year-olds in the United Kingdom,^9^ while the United States has no organized screening program and requires referral by a medical practitioner.^10^ Other factors that may contribute to these differences are hormone replacement therapy use and oral contraceptive use. When modeling incidence/mortality trends, these factors are known as both period and cohort effects. Given these apparent age-related differences, it may be misleading to estimate future breast cancer mortality patterns without accounting for age-related time trends in mortality.

In this study, we used a recently developed forecasting method^11^ to (1) compare the time trends of age-specific breast cancer mortality for the United States and England–Wales and (2) predict future rates of age-specific breast cancer mortality for the United States and England–Wales. The forecasting method we use predicts the entire age-mortality relationship through time, and does not simply rely on the most recent data. The good forecasting performance of the present models in other contexts^12^ suggests that the models are likely to increase the accuracy of predictions in the present context as well. To the best of our knowledge, this is the first study to incorporate the effect of age-specific trends over time on breast cancer mortality when estimating future trends of age-specific breast cancer mortality in England–Wales and the United States.

## METHODS

### Data

Annual age-specific breast cancer mortality data for England and Wales from 1950 through 2003 were obtained from the Office of the National Statistics.^3^ US mortality data from 1950 through 2001 were extracted from the World Health Organization mortality database.^13^ Data for breast cancer are designated by ICD-6 and -7 code 170, ICD-8 and -9 code 174, and ICD-10 code C50. The data are available in 5-year age groups: 45–49, 50–54, 55–59, 60–64, 65–69, 70–74, 75–79, and 80–84 years.

### Statistical analysis

Breast cancer mortality was observed annually as a function of age, defined as the midpoint of the age groups. For each year, we plotted the age and mortality associations, which we refer to as mortality-age curves. We then took the log of the mortality rate (our outcome) at each midpoint of age for each year and used functional data analysis (FDA) techniques^14^ to model these annual mortality-age curves collectively as a functional time series. In these models we first assumed an underlying smooth function that we are observing with error (see the Appendix for details). We then used nonparametric regression techniques to estimate the smooth curves.^15^^,^^16^

Next, we took these smooth curves as our functional observations and fit functional data analysis models. We followed the estimation procedure of Hyndman and Ullah^11^ and applied functional principal components decomposition^17^ to the smooth curves, because this approach produces a small number of basis functions, enables informative interpretations, and yields coefficients that are uncorrelated with each other.

To predict future mortality, we forecast each coefficient in the models using a univariate time series model. We multiplied these forecasts with the basis functions in the FDA models, resulting in forecasts of mortality age curves through time. We then used exponential smoothing state space models to compute the forecasts^18^ and construct prediction intervals around our predictions.^19^ We used the Mean Integrated Squared Forecasting Error (see Appendix) to evaluate the accuracy of the estimated predictions of future mortalities. For the US and England–Wales mortality data we estimated 20-year predictions using exponential smoothing state space models with damping.^18^ All statistical analyses were performed using R version 2.3.1.

## RESULTS

**Figure 1.**
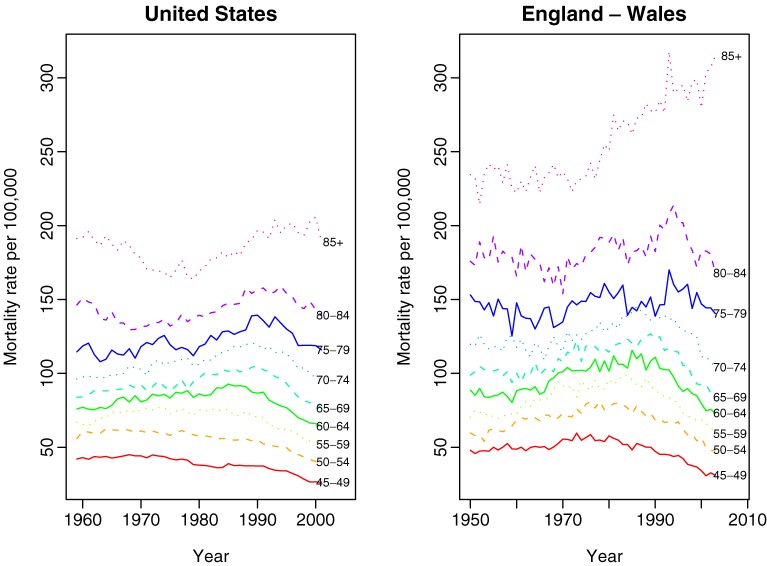
Observed breast cancer mortality trends by age group for the United States (left) and England–Wales (right).

Figure 1 displays the observed breast cancer mortality time trends by age for the United States (per 100 000 women) from 1959 through 2001 (left) and for England–Wales (per 100 000 women) from 1950 through 2003 (right). For the United States, mortality trends for middle-aged women (45–54 years) have continued to decline since 1980. Mortality trends for women between 60 and 84 years of age increased in the 1980s and subsequently declined in the 1990s. The observed pattern of mortality is similar for England–Wales. Mortality trends for women aged 45 to 74 years slightly increased between 1950 and 1980, but declined thereafter. Mortality trends for women aged 75 to 79 years and 80 to 84 years fluctuated during the study period. However, as was the case for the other age groups, there was an overall decline in mortality during the 1990s.

Under our model, an adequate fit (as determined by the MISE) was a functional regression model with 2 basis functions for the US data; for England–Wales, it was a functional regression model with 3 basis functions. The first basis function accounts for 68.4% of variation around the mean log mortality curve for the United States and 71.5% of variation around the mean log mortality curve for England–Wales. For England–Wales, β_1_ showed an increase in mortality for all age groups between 1950 to 1980, followed by a rapid decline until 2000. Similar interpretations can be made for the United States.

**Figure 2.**
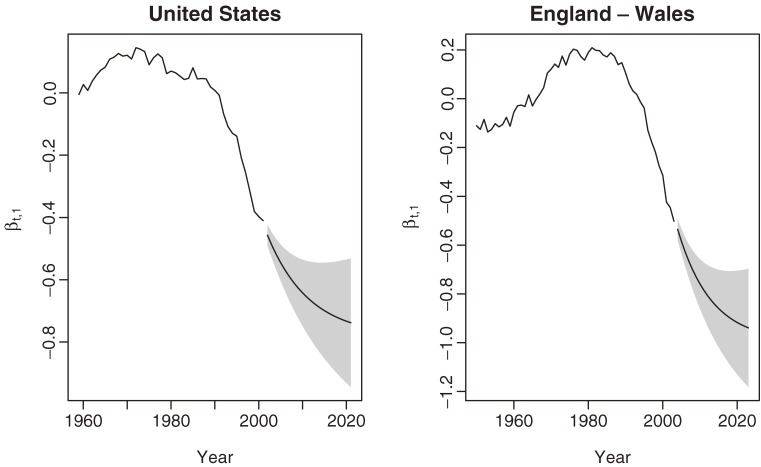
Twenty-year mortality predictions for the United States (left) and England–Wales (right) using a damped trend exponential smoothing model. The y-axis represents the estimated coefficient of the first basis function. The shaded region gives the 80% prediction interval.

Twenty-year projections of the first basis function, which controls the overall change in trend of breast cancer mortality, are shown in Figure 2, along with 80% prediction intervals, for (1) the United States from 2002 through 2021 and (2) England–Wales from 2004 through 2023. The y-axis represents the coefficients associated with the first basis function, ie, the overall change in trend of breast cancer mortality. Predictions from the models suggest that overall crude mortality rates for both countries will decline more slowly than during the 1990s. These predictions assume no changes or advances in treatment.

Figures 3a and 3b display 20-year predictions of age-specific breast cancer mortality for both regions Mortality trends are expected to decline for all women, with the greatest decline among women aged 60 to 70 years, whereas estimated predictions suggest relatively stable trends for women older than 75 years.

**Figure 3a.**
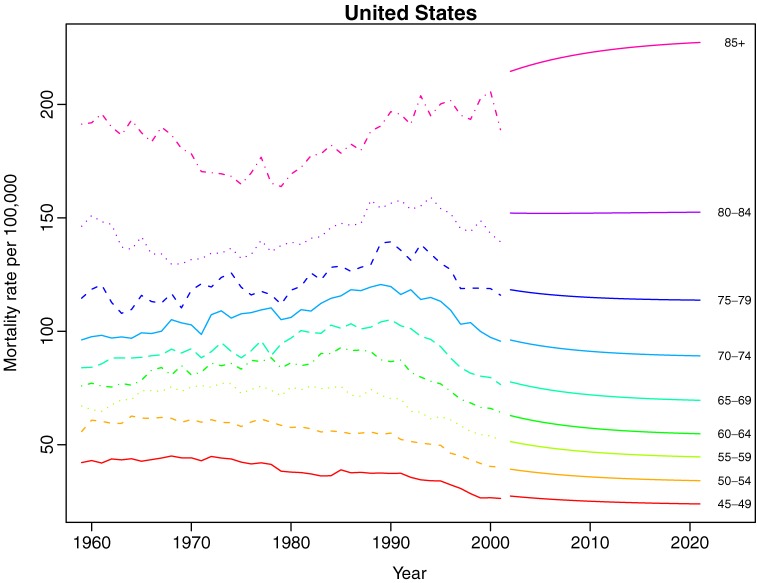
Estimated 20-year predictions of age-specific breast cancer mortality for the United States.

**Figure 3b.**
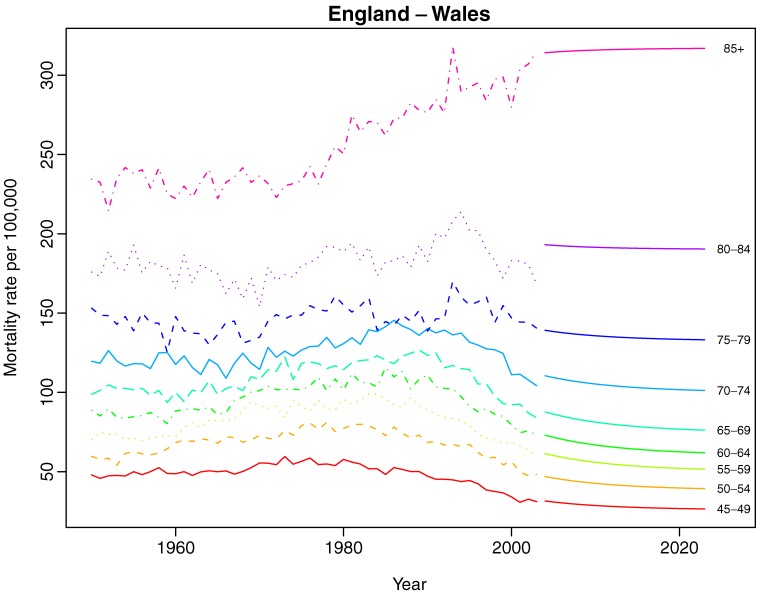
Estimated 20-year predictions of age-specific breast cancer mortality for England–Wales.

**Figure 4.**
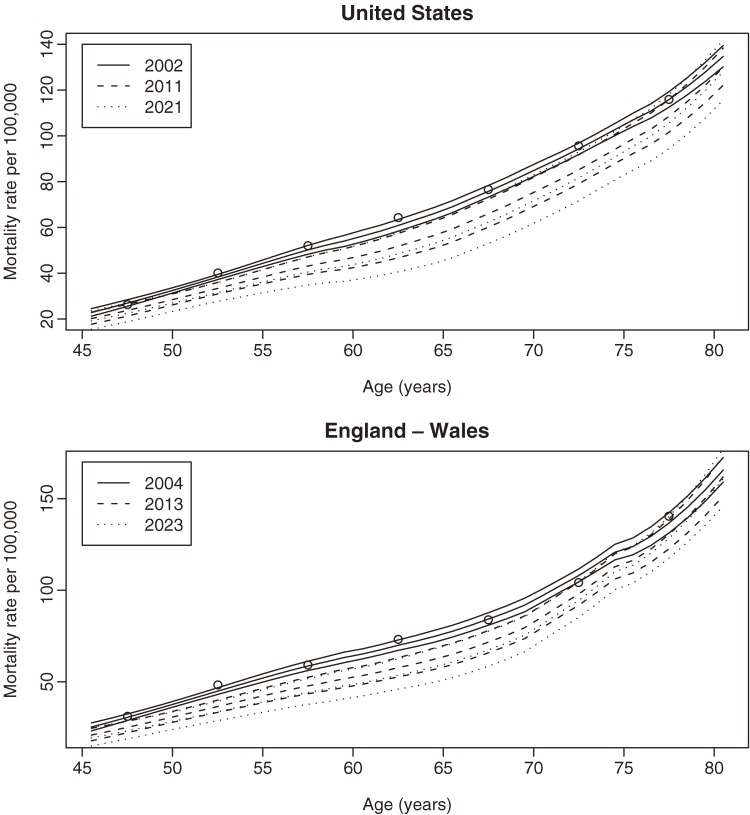
Estimated 1-, 10-, and 20-year age-specific predictions for the United States (top) and England–Wales (bottom). The circles represent actual values.

To evaluate the accuracy of the predictions, we estimated 1-, 10-, and 20-year age-specific mortality predictions with 80% prediction intervals for both regions (Figure 4). The estimated predictions had very narrow prediction bands. For example, 20-year predictions for 60-year-old women in England–Wales had an 80% error margin of less than 10 deaths per 100 000 women.

We examined the residual of the functional fits using image plots (data not shown). These images showed no evidence of lack of fit and suggest that the few remaining birth cohort trends are of no concern.

## DISCUSSION

Using an innovative forecasting method, our 20-year projections suggest a continuing decline in breast cancer mortality for both England–Wales and the United States. There has been considerable debate regarding the primary factors responsible for the decline in mortality observed in England–Wales, and to a lesser extent in the United States.^20^^,^^21^ In England–Wales, the decline in mortality since the early 1990s has often been attributed to better treatment practices and the widespread use of tamoxifen.^20^ At the end of the 1990s and more recently, screening is also believed to have played a role, but there are doubts as to whether it had a major impact on mortality in the early 1990s, when the sharpest decline occurred.^22^^,^^23^ Since 1988, the United Kingdom has had an organized mammographic screening program in place for women aged 50 to 70 years,^24^ whereas in the United States screening is more ad-hoc and is recommended for women over 40 years of age.^21^ It is therefore possible that screening contributed to the greater decline in women older than 75 years in the United Kingdom, but not in the United States. Other factors, such as the rapid increase in hormone therapy use in the 1990s and the subsequent decline in its use after the release of the Women’s Health Initiative trial findings,^25^ may have contributed to the continuing decline in breast cancer mortality for England–Wales. The reduction observed in the United States has been attributed to early detection through screening, combined with the use of adjuvant chemotherapy and tamoxifen for patients with all stages of breast cancer.^26^

This study has a number of important strengths. First, we have presented an alternative modeling approach to the classic age-period-cohort (APC) models. Here, mortality rates are regarded as smooth functions of age, and the shape of the mortality-age relationship is allowed to change over time. Our modeling and forecasting approach is appealing because of the few assumptions required and the visual character of the projections. Second, unlike most other studies, we estimated prediction intervals for future mortality rates. Prediction intervals are necessary to accommodate uncertainty in the mathematical structure of the model and variation in the future mortality rate.^27^ The prediction intervals from the functional forecasting models are narrow for both the overall and age-specific breast cancer mortality rates in England–Wales and the United States, suggesting that the models have captured the stochastic and dynamic properties of the data. As expected, prediction intervals widened as the forecast horizon increased.

A number of limitations should be considered when interpreting the long-term mortality trends reported here. First, changes in coding practices, the accuracy of death reporting throughout the period of data collection, revisions of ICD codes, and the combining of subsites into one major site may affect our interpretation of cancer trends^28^; however, for breast cancer, the general consensus is that the consistency of the data has been reasonable.^29^

Second, birth cohort effects due to changes in the underlying risk factors were not included in the models and these may alter the shape of the age-mortality distribution.^27^ Birth cohort trends for US women born from 1880 through 1915 would have affected the estimates and resulted in higher-than-expected projections for older women. At present, the decreasing birth cohort trends for baby boomers have not had their full impact on the decline in future mortality rates in the United States. In a study of the mortality benefits of screening in England–Wales,^30^ birth cohort effects were similar for all ages before the introduction of screening. The smoothing process used in our modeling may reduce the variation attributable to birth cohort effects. Furthermore, any remaining birth cohort effects will be saturated in patterns of variation over time. Nevertheless, birth cohort trends are an important aspect of modeling mortality/incidence trends and we are currently developing models which incorporate cohort effects and other variables that may influence age-related changes in mortality (or incidence).

In summary, we presented a new modeling and forecasting technique to model and estimate future trends in breast cancer mortality. At present, much of the modeling and prediction of mortality trends uses APC models. Here, we presented an alternative framework, which can predict entire age-mortality curves for each period, to estimate predictions, thereby enhancing the accuracy of the predictions. In their current form, these models will be most useful for modeling and projecting the future trends of diseases for which there has been very little advancement in treatment and minimal birth cohort effects, such as cancers of the pancreas and brain.

## APPENDIX

We used functional data analysis techniques^14^ to model annual mortality-age curves collectively as a functional time series. Specifically, let $y_{t}(x)$ denote the log mortality at age x for year t. We assume that there is an underlying smooth function $f_{t}(x)$ that we are observing with error. Thus, we observe the functional time series $\left\{x_{i},y_{t}(x_{i})\right\}$, t=1,…,n, i=1,…,p, where

$$y_{t}(x_{i})=f_{t}(x_{i})+\sigma_{t}(x_{i})\varepsilon_{t,i},$$ (1)

where $\left\{\varepsilon_{t,i}\right\}$ are independent and identically distributed random variables with zero mean and unit variance, and $\sigma_{t}(x_{i})$ describes how the amount of error varies with x.

The smoothed curves $\left\{f_{t}(x)\right\}$ are estimated using nonparametric regression techniques, such as penalized regression splines^15^ and Loess curves.^16^ The n smoothed curves are our functional observations, $\left\{f_{t}(x)\right\}$, where $x_{1}<x<x_{p}$ and t=1,…,n. Erbas, Hyndman, and Gertig^19^ and Hyndman and Ullah^11^ proposed the following model for the smoothed curves:

$$f_{t}(x)=\mu (x)+\sum_{k=1}^{K}\beta_{t,k}\phi_{k}(x)+e_{t}(x),$$ (2)

where μ(x) is a measure of the mean log mortality across years, $\left\{\phi_{k}(x)\right\}$ is a set of orthonormal basis functions, and each $\beta_{t,k}$ is a univariate time series. We follow their estimation procedure by computing $\left\{\phi_{k}(x)\right\}$ using functional principal components decomposition^17^ applied to the smoothed curves $\left\{f_{t}(x)\right\}$, as this approach produces a small number of basis functions, enables informative interpretations, and yields coefficients that are uncorrelated with each other. For a given value of K, we choose a model with basis functions $\left\{\phi_{k}(x)\right\}$ that minimize the mean integrated squared error (MISE): $=\frac{1}{n}\sum_{t=1}^{n}\int e_{t}^{2}(x)dx$

To make predictions of future mortality rates, we forecast each coefficient $\left\{\beta_{t,k}\right\}$ using a univariate time series model. We multiply these projections by the basis functions, resulting in projections of mortality curves $f_{n+h}(x)$, h=1,2,…. We then use exponential smoothing state space models to compute the forecast^18^ and construct prediction intervals around our predictions.^19^ The Mean Integrated Squared Forecasting Error: $(\mathrm{MISFE}(h))=\frac{1}{n-m+1}\sum_{t=m}^{n}\int {\left[y_{t+h}(x)-{\hat{f}}_{t,h}(x)\right]}^{2}dx$ is used to evaluate the accuracy of the estimated predictions of future mortalities.
